# Occurrence of *Escherichia coli* non-susceptible to quinolones in faecal samples from fluoroquinolone-treated, contact and control pigs of different ages from 24 Swiss pig farms

**DOI:** 10.1186/s40813-021-00209-y

**Published:** 2021-04-02

**Authors:** Morena Amsler, Katrin Zurfluh, Sonja Hartnack, Xaver Sidler, Roger Stephan, Dolf Kümmerlen

**Affiliations:** 1 Department of Farm Animals, Division of Swine Medicine, Vetsuisse Faculty, University of Zurich, Winterthurerstrasse 260, 8057 Zurich, Switzerland; 2 Institute for Food Safety and Hygiene, Vetsuisse Faculty, University of Zurich, Winterthurerstrasse 272, 8057 Zurich, Switzerland; 3 Section of Epidemiology, Vetsuisse Faculty, University of Zurich, Winterthurerstrasse 270, 8057 Zurich, Switzerland

**Keywords:** Fluoro−/quinolone resistance, Pigs, *Escherichia coli*, Faecal samples, Environmental samples

## Abstract

**Background:**

Despite their indispensability in human medicine, fluoroquinolones (FQ) are used for the treatment of bacterial infections in farm animals which increases the risk of transferring FQ-resistant bacteria into the environment and via the food chain to humans. The objectives of this observational study were to follow-up of the presence of quinolone non-susceptible *Escherichia coli* (QNSE) qualitatively and quantitatively in faecal samples of pigs at four time points (2 weeks old, 4 weeks old, 2 weeks post weaning and during fattening period). Moreover differences between groups of FQ-treated pigs, pigs with contact to treated pigs and control pigs were investigated. Additionally, quinolone and FQ resistance of *Escherichia coli* isolates of the faecal samples were investigated by determining minimum inhibitory concentrations (MICs).

**Results:**

40.9% of 621 fecal samples contained QNSE. Proportion of samples with detectable QNSE from treated and contact pigs did not differ significantly and were highest in piglets of 2 and 4 weeks of age. However, the proportions of samples with QNSE were significantly lowest in control pigs (7/90; 7.8%; CI = 3.5–14.7%) among all groups. Also, the number of colony-forming units was lowest in both weaners and fattening pigs of the control group compared to treated and contact groups. Following CLSI human breakpoints, in total, 50.4% out of 254 isolates in faecal samples were intermediate or resistant to ciprofloxacin.

**Conclusions:**

QNSE were present in faeces of pigs independent of age or FQ background but significantly less were found in pigs from farms without FQ usage. Due to the long half-life of FQ, it is likely that only a prolonged absence of fluoroquinolone treatments in pig farming will lead to a reduced frequency of QNSE in the farm environment. Solutions need to be found to minimise the emergence and transfer of quinolone and FQ-resistant bacteria from treated pigs to contact pigs and to farms without FQ usage.

**Supplementary Information:**

The online version contains supplementary material available at 10.1186/s40813-021-00209-y.

## Background

Quinolones, e.g. nalidixic acid (NA), are synthetic antimicrobial agents introduced for the first time in 1963. Chemical modifications enabled fluoroquinolones (FQ) to work against a wide spectrum of bacteria, including *Enterobacteriaceae*, gram-positive bacteria and anaerobes, in many different body tissues.

According to the classification published by the World Health Organization (WHO), FQ are part of the highest priority critically important antimicrobials (HPCIAs) due to their essential role in treating patients suffering from zoonotic diseases, e.g. salmonellosis and campylobacteriosis, or protecting neutropenic patients from septicemia [1, 2].

In Europe, Canada and Japan resistance rates of porcine pathogenic FQ-resistant *E. coli* isolated from swine lie between 0 and 39% [3, 4]. This is in contrast to China and Brazil who report very high resistance rates (81.0 and 54.4%) in porcine pathogenic and commensal *E. coli* from swine [5, 6]. In pathogenic and commensal *E. coli* isolated from Swiss pigs (sows, weaners and fattening pigs) a low rate of ciprofloxacin (CIP) resistance (< 2%) was reported [7, 8].

The number of humans infected with FQ resistant bacteria has increased since the introduction of FQ into veterinary medicine. An association between the prevalence in swine herds or poultry flocks and the number of diseased patients was shown [9, 10]. Restricted prescriptions and therapeutic guidelines for veterinarians should promote a responsible and sustainable handling of antimicrobials since the occurrence of FQ-resistant bacteria and any transfer from livestock to humans could impair human health [11, 12]. Slaughter processes and kitchen hygiene are two crucial points in the transfer of pathogenic bacteria [13, 14] and also of resistant bacteria. Nevertheless, animal transport, liquid manure spread onto croplands and dust from farms are other considerable transmission pathways in livestock and between animals and human beings [15–17].

Current research investigating FQ resistance rates and transfer between FQ-treated and FQ-untreated pigs is contradictory when comparing two experimental studies. According to the first report, in both groups (housed in the same room) commensal *E. coli* with minimal inhibitory concentration (MIC) ≥ 4 mg/ml enrofloxacin were detected [18]. However, in another recently published study, during and 42 days after FQ treatment, no CIP-resistant *E. coli* (MIC ≥4 mg/ml enrofloxacin) were observed either in the treated, untreated contact groups o the control group (housed in a separate room) [19]. Depending on age, fattening pigs showed lower resistance rates compared to pigs of younger ages [20, 21].

In the present observational study, QNSE were longitudinally monitored in faecel samples of pigs treated with FQ, in contact pigs without treatment, and in a control group at four different time points. The study aimed to quantitatively and qualitatively evaluate whether the occurrence of QNSE differed between the study groups and at the different timepoints. Additionally, MICs of NA and CIP were determined for *E.coli* isolated from the faecal samples and differences between study groups were investigated. Improved understanding of the relationship between FQ use and the emergence of FQ resistance will help reduce the risk of transfer to humans and subsequent impairment of human health.

## Methods

### Study population

Sampling was performed on 24 Swiss pig farms between May 2017 and May 2018: Thirteen farms were part of a sow-pool-system (SPS) with a total of 1′200 sows, in which sows were transported between farms housing them either during the mating, gestation or farrowing period. The size of the study farms ranged from 13 to 75 sows. Table 1 provides more detailed information concerning the structure of the study farms.

**Table 1:** Group formation and distribution of different farm structures

group		code	FQ treatment of sows	FQ treatment of piglets	farrow-to-finish	farrowing	farrow and rearing	rearing and finishing	fattening
G1	Trt	S + P-	+	–	1	2 (SPS)	1 (SPS)	1 (SPS)	7 (7 SPS)
G2	Ctat	S-P-	–	–					
G3	Trt	S-P+	–	+			2 (1 SPS)		6 (5 SPS)
G4	Ctat	S-P-	–	–					5 (5 SPS)
G5	Ctrl	No FQ usage on farm	–	–	2		1		3 (1 SPS)
**TOTAL**					**3**	**2 (SPS)**	**4 (2 SPS)**	**1 (SPS)**	14 (8 SPS)*

*S* sows, *P* piglets, + = FQ treatment, - = no FQ treatment, *SPS* part of a sow-pool-system, *Trt* treated, *Ctat* contact, *Ctrl* control

Inclusion criteria for study farms was a regular FQ usage restricted to either piglets or sows (Table 1). Additionally, farms with no use of FQ in any age category for at least three to 34 months were included to compare the dissemination of quinolone non-susceptible *E. coli*. Potential study farms were reported by the pig trading company and definitive study farms were then randomly selected.

The main indications for FQ treatment in sows and piglets were either postpartum dysgalactia syndrome (PPDS) in sows or septic arthritis or diarrhea in piglets. Treatments were carried out by the farmers following veterinarians prescriptions including single and multiple FQ treatments (according to the drugs’ summary of products characteristics (SPC). Parenteral treatment in sows included either Baytril®5% (enrofloxacin), 2.5 mg/kg SID, or Marbocyl®10% (marbofloxacin), 2 mg/kg SID. Treatments in piglets included either Marbocyl®2% (marbofloxacin) parenteral, 2 mg/kg SID or Baytril®0.5% (enrofloxacin) orally, 1.7 mg/kg SID). Two piglets received a second FQ treatment after weaning (Baytril®0.5% (enrofloxacin), 1.7 mg/kg SID) but the remaining sows and their progeny did not receive any additional FQ treatment during the study apart from the initial FQ treatment. Other reported antimicrobials used during the study were sulfadoxin-trimethoprim (parenteral in sows), amoxicillin, benzylpenicillin and in combination with streptomycin (parenteral in piglets), colistin, sulfadimidine-sulfathiazole-trimethoprim, chlortetracycline and chlortetracycline-sulfadimidin-tylosin (oral during weaning or fattening).

Sampled pigs were divided into five groups. In two groups (G1 and G3, summarised as Trt) either the sows or the piglets were treated with fluoroquinolones (Table 1). On every farm, sampling of contact piglets (ctat) was performed forming group G2 (contact piglets to G1) and G4 (contact to G3). Contact pigs are summarised as pigs not treated with FQs but held in the same group with FQ-treated pigs, i.e. having direct contact, or pigs not treated with FQs but held in the same room or farm, i.e. having indirect contact. Control pigs from farms without FQ use for more than 3 months belonged to the fifth group (G5 = ctrl: sows and piglets from farms without FQ usage). Information on group formation and farm structure are summarised in Table 1. Study designers did not have any influence on the distribution of group animals from the farrowing to the fattening units. Thus, one fattening unit received pigs belonging to the third group (G3) but no pigs belonging to the fourth group (G4).

### Faecal sampling

Farrowing farms were contacted after the sows’ expected delivery date. When FQ treatment was reported, farms were visited approximately 2 weeks after farrowing. Sampling was performed in two different steps in the farrowing units: In farms with FQ use in sows three 2 weeks old piglets from every sow (treated or untreated) were randomly picked. Pooled samples of approximately 1–5 g faeces of the three piglets of each sow were taken rectally or during defaecation. This procedure was used to ensure sufficient sample material for the following laboratory procedures.

In farms with FQ use in piglets, we were also able to collect individual faecal samples of the same amount described above (1–5 g). Contact and treated piglets were picked from the same litter if piglets were suffering from septic arthritis. Since farmers performed metaphylaxis (treating all piglets) in litters suffering from diarrhoea, a different litter was selected for sampling contact piglets. Gloves were changed after each sample to avoid cross-contamination.

A numbered ear tag of contrasting colour in the left ear and the four-digit number of the Swiss animal movement database in the right ear ensured group and individual identification for the following samplings.

Because of a low prevalence of CIP resistance in *E. coli* from Swiss pigs and a study recommending pooled samples only if the prevalence of resistance is > 2% we preferred to collect single faecal samples in the following samplings to record the pigs’ individual courses [7, 8, 22].

**Fig. 1:**
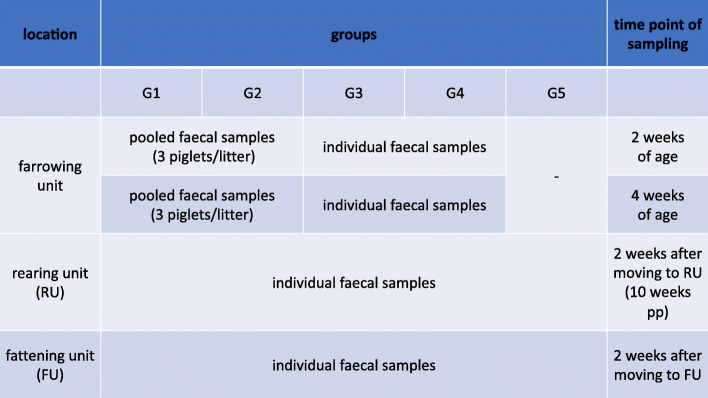
Protocol for sampling of study animals

Sampling was timed by the dates when pigs were moved to another facility to assess quinolone susceptibility status in every animal before and after movement: in the farrowing unit, piglets were resampled at 4 weeks of age shortly before moving to the rearing unit. Subsequently, faeces were collected at the end of rearing around 10 weeks of age (weaners) and at least 2 weeks after moving to the fattening unit (fattening pigs at an age of around 12 weeks). Because there had been no use of FQ for more than 3 months in the farrowing unit we forewent collecting piglet faeces from group 5 (Fig. 1). Due to subsequent processing collected faeces were individually kept in a stool tube and stored at − 20 °C on the same day.

### Laboratory methods

Samples were thawed at 7 °C overnight and tested semi-quantitatively for the presence of non-susceptible *E. coli* to quinolone. Approximately 1 g or 1 ml of sample was diluted 1:10 with 0.85% saline solution and homogenised in a Stomacher® (Seward Stomacher® 400 Laboratory Blender BA 7021, West Sussex, UK). The homogenate was streaked in different dilutions by the pour plate method on selective Rapid-*E. coli* 2 agar plates (Biorad®, Munich, Germany) supplemented with 8 mg/L NA, 10 mg/L vancomycin and 5 mg/L amphotericin B. After overnight incubation at 37 °C presumptive positive *E. coli* colonies (β-D-glucuronidase and β-D-galactosidase positive, presented as purple and round colonies) were counted. Plates with a massive *E. coli* growth where counting was impossible, were given an approximate number of 100′000 counts per plate. From each sample one *E. coli* colony was randomly selected for further investigations. Isolates underwent disk diffusion (DD) susceptibility testing including antimicrobials (Becton Dickinson and Company, Sparks, MD USA) NA (NA30) and CIP (CIP5). In isolates intermediate and resistant to NA, the MIC (μg / ml) to NA and CIP was assessed using ETEST® strips (BioMérieux, Marcy l’Etoile, France). Performance and interpretation of susceptibility testing followed the guidelines of the Clinical and Laboratory Standards Institut [23]. Slight deviations are described as follows: For colony suspension broth culture method (using tryptic soy broth) was used and broths with selected *E. coli* colonies were incubated over night at 37 °C for approximately 13 to 20 h. Mueller-Hinton agar were streaked using 0.5 McFarland standard suspension. After streaking lids were not left ajar but inverted for a couple of minutes before applying discs or ETEST® strips and incubated overnight at 37 °C for approximately 13 to 20 h. Quality control was performed in every new batch of Mueller-Hinton agar plates using *Escherichia coli* ATCC® 25922 with results within the expected ranges listed. Due to a lack of animal-specific breakpoints, MICs were interpreted according to human pathogen specific breakpoints published by CLSI [23]. MIC values of NA and CIP were defined as intermediate resistant if lying between > 16 μg/ml and < 32 μg/ml and > 0.25 μg/ml and < 1 μg/ml, respectively. Moreover, isolates were differentiated into wild-type (WT) and mutant (M) by epidemiological cut-offs (ECOFFs *Escherichia coli*; ECOFF_Nalidixic acid_: 8 mg/L, ECOFF_ciprofloxacin_: 0.064 mg/L) published by The European Committee on Antimicrobial Susceptibility Testing (EUCAST) [24].

### Data analysis and statistical evaluation

#### Descriptive statistics and confidence intervals (95% CI)

Descriptive and inferential statistics were performed in IBM® SPSS® Statistics for Macintosh Version 25.0 and the software program R Version 3.5.1 [25]. QNSE counts were expressed as log CFU/g or ml except for zero QNSE counts (expressed as 0 CFU/g or ml). The number of samples with detectable QNSE divided by the total number of tested samples is described as proportion (%). FQ susceptibility proportions (%) of isolated *E. coli* are described. Binomial and multinomial confidence intervals (95% CI) were obtained using the Jeffreys approach [26] and MultinomCI from the package by DescTools [27]. Non-overlapping confidence intervals were considered to be significantly different.

#### Mixed-effects models

Hurdle Poisson mixed-effects models were used to assess if the counts of quinolone non-susceptible *E. coli* (QNSE) in weaners and fattening pigs (using the original size scales, for further information see Additional file 1) differed between the groups Trt (treated pigs; G1 and G3), Ctat (contact pigs; G2 and G4) and Ctrl (control pigs; G5, FQ free) with the package GLMMadaptive [28]. To adjust for potential clustering, the rearing units were considered as a random effect. The hurdle models comprise of two parts: the zero-count part (presence or absence of QNSE) is considered to follow a binomial distribution (logistic regression) and the positive-count part (counts of QNSE) is treated as a Poisson distribution. Additionally, models with a zero-inflated negative binomial distribution were also tested. Model fit was assessed by likelihood ratio tests. QNSE count results from the mixed-effects models (original size scales) were transformed in log CFU/g.

## Results

### Demographic data of faecal samples (groups and age categories)

In this study, we included 218 pigs of which eleven pigs could not be followed up until the fattening unit (three pigs died, one pig was euthanised, seven pigs were undetectable). Overall, 621 faecal samples (116 faecal samples from 2 weeks old piglets, 104 from 4 weeks old piglets, 206 from pigs during rearing and 195 from fattening pigs) were tested and used for further analysis.

### Proportions of samples with detectable QNSE

In 40.9% (254/621) of all faecal samples, quinolone non-susceptible *E. coli* (QNSE) were detected. MICs for NA and CIP and counts of colony-forming units of each of the isolates of faecal samples are shown in Additional file 1. Two weeks old piglets showed the highest proportion of samples with QNSE, followed by 4 weeks old piglets, fattening pigs and weaners (Table 2). Confidence intervals of the QNSE proportion of all *E. coli* significantly differed between piglets (2 and 4 weeks old) and weaners and between piglets and fattening pigs, respectively. The proportion of QNSE was lower in samples of weaners compared to samples of fattening pigs but 95% CI did not differ significantly.

**Table 2:** Quantitative detection and proportions of samples with detectable quinolone non-susceptible *Escherichia coli* (QNSE)

	Groups					
	G1	G2	G3	G4	G5	TOTAL
**FARU**						
**2w p.p.**	94.4% (17/18), p	100.0% (18/18), p	92.5% (37/40)	92.5% (37/40)		94.0% (109/116)
	CI = 76.8–99.4%	CI = 87.1–100.0%	CI = 81.3–97.9%	CI = 81.3–97.9%		CI = 88.5–97.3%
	x_0_ = 1	x_0_ = 0	x_0_ = 3	x_0_ = 3		x_0_ = 7
	ø = 7.0	ø = 6.7	ø = 7.1	ø = 5.8		ø = 6.9
	m = 6.6	m = 5.8	m = 5.6	m = 4.6		m = 5.7
**4w p.p.**	94.1% (16/17), p	91.7% (11/12), p	72.5% (29/40)	91.4% (32/35)		84.6% (88/104)
	CI = 75.6–99.4%	CI = 67.1–99.1%	CI = 57.4–84.5%	CI = 78.8–97.6%		CI = 76.7–90.6%
	x_0_ = 1	x_0_ = 1	x_0_ = 11	x0 = 3		x_0_ = 16
	ø = 6.4	ø = 6.4	ø = 5.4	ø = 5.1		ø = 5.9
	m = 5.1	m = 6.3	m = 3.8	m = 3.8		m = 4.3
**RU**	13.3% (6/45)	15.4% (6/39)	10.8% (4/37)	2.5% (1/40)	11.1% (5/45)	10.7% (22/206)
	CI = 5.7–25.5%	CI = 6.6–29.0%	CI = 3.7–23.7%	CI = 0.2–11.1%	CI = 4.3–22.7%	CI = 7.0–15.5%
	x_0_ = 39	x_0_ = 33	x_0_ = 33	x0 = 39	x_0_ = 40	x_0_ = 184
	ø = 2.3	ø = 3.1	ø = 1.0	ø = 3.4	ø = 1.7	ø = 2.9
	m = 0.0	m = 0.0	m = 0.0	m = 0.0	m = 0.0	m = 0.0
**FU**	23.8% (10/42)	14.3% (6/42)	29.6% (8/27)	25.0% (9/39)	4.4% (2/45)	15.6% (35/195)
	CI = 12.9–38.2%	CI = 6.1–27.1%	CI = 15.1–48.3%	CI = 12.0–38.0%	CI = 0.9–13.6%	CI = 13.0–23.8%
	x_0_ = 32	x_0_ = 36	x_0_ = 19	x0 = 30	x_0_ = 43	x_0_ = 160
	ø = 2.9	ø = 2.3	ø = 2.2	ø = 2.1	ø = 1.9	ø = 2.5
	m = 0.0	m = 0.0	m = 0.0	m = 0.0	m = 0.0	m = 0.0
**TOTAL**	40.2% (49/122)	36.9% (41/111)	54.2% (78/144)	51.3% (79/154)	7.8% (7/90)	40.9% (254/621)
	CI = 31.7–49.1%	CI = 28.3–46.2%	CI = 46.0–62.2%	CI = 43.4–59.2%	CI = 3.5–14.7%	CI = 37.0–44.9%
	x_0_ = 73	x_0_ = 70	x_0_ = 66	x0 = 75	x_0_ = 83	x_0_ = 367
	ø = 6.3	ø = 6.1	ø = 6.6	ø = 5.3	ø = 1.8	ø = 6.2
	m = 0.0	m = 0.0	m = 100.0	m = 100.0	m = 0.0	m = 0.0

Proportions of samples with QNSE and corresponding 95% confidence intervals (CI), x_0_ = number of samples with zero quinolone non-susceptible *E. coli* detected, mean (= ø) and median (= m) log colony forming unit per gram faeces (log CFU/g) of quinolone non-susceptible *E.coli* in faecal samples from pigs of different age and group, median = 0.0 were expressed in colony forming unit per gram faeces (CFU/g), *FARU* farrowing unit, *2/4w p.p.* two and four weeks postpartum, *RU* rearing unit, *FU* fattening unit, *p* pooled samples

QNSE also were found in weaners and fattening pigs from farms without FQ use (G5 = 7.8%, 7/90). In total, the proportions of samples with QNSE was significantly lower in control pigs than in pigs of group 1–4. All seven isolates originated from a total of 29 samples from one farm on which the last FQ usage was carried out 3 months ago before sampling. The last FQ usage of all other farms from G5 had been carried out six to 34 months before sampling.

There were no significant differences in the proportions of samples with detectable QNSE among any of the five groups comparing within any specific time point.

### Quantitative detection of QNSE

From piglets to fattening pigs there is a decrease in mean and median log counts of QNSE colony forming units per gram faeces (log CFU/g faeces). Highest means were detected in 2 weeks old piglets of G3 and G1. In the rearing and fattening unit, the lowest means were observed in G3 and G5 (Table 2).

### Quantitative and qualitative detection of QNSE – hurdle models

The lowest means of colony-forming units per gram faeces in weaners and fattening pigs were observed in the control group (Ctrl) compared to groups with treated (Trt) and contact (Ctat) pigs. The Poisson hurdle model with random effects indicated significant differences in the count part, i.e. for QNSE counts significant differences between groups were observed in weaners and fattening pigs (highlighted with an asterisk in Table 3). Control pigs showed the significantly lowest counts in both weaners and fattening pigs. In the zero-part, i.e. detection of QNSE versus no detection of QNSE, no significant differences were observed between the three groups in weaners. In fattening pigs, values showed large standard errors and therefore were not plausible. Effect sizes for the zero-part represent odd ratios.

**Table 3:** Quantitative and qualitative detection of quinolone non-susceptible *Escherichia coli* (QNSE) – Hurdle

		group		
		Trt	Ctat	Ctrl
**weaners**	**mean**	2.1	3.3	1.7
	**x**_**0**_	72	72	40
	**count part (CI 95%)**	1.888 (1.877–1.899)*	3.757 (3.754–3.760)*	1.707 (1.689–1.725)*
	**zero part (CI 95%)**	1.522 (1.013–2.033)	1.519 (1.009–2.029)	1.497 (0.991–2.003)
**fattening pigs**	**mean**	2.8	2.2	1.9
	**x**_**0**_	51	66	43
	**count part (CI 95%)**	3.378 (3.373–3.383)*	2.800 (2.791–2.809)*	1.936 (1.922–1.949)*
	**zero part (CI 95%)**	4.3E-04# (8.6E-05-2.2E-03)	1.1E-03# (2.2E-04-5.2E-03)	1.3E+ 04# (3.1E+ 03–5.7E+ 04)

Hurdle models: x_0_ = number of samples with zero QNSE detected, mean = mean of colony forming units (CFU) per gram (g) faeces, count part, zero part = hurdle models with count and zero part and confidence intervals (CI 95%) in log CFU/g faeces. Trt = treated group (G1 and G3), Ctat = contact group (G2 and G4), Ctrl = control group (G5), * = significant values (not overlapping confidence intervals), # = indicates not convertible hurdle models with large and non-useful standard errors

### MIC data of faecal samples

#### MICs of NA

MICs of NA were tested in 254 randomly selected isolates from each group of which the MIC 50 and 90% of NA were > 256 μg/ml. Overall, the range of MICs was 24 - > 256 μg/ml. Except for three isolates, all of the piglets isolates from G3 and G4 achieved MICs between 256 and > 256 μg/ml. Isolates’ MICs of G1 and G2 showed a wider range (24 - > 256 μg/ml) in piglets. Observing all ages isolates of G3 (*n* = 78), G4 (*n* = 79) and G5 (*n* = 7) reached the same MIC 50 and 90% (> 256 μg/ml). Further information concerning the MICs of NA can be obtained from Additional file 2.

Out of all faecal isolates 98.4% (250/254) were resistant to NA and four isolates (1.6%) showed intermediate resistant results according to Clinical and Laboratory Standards Institute (CLSI) guidelines. 100% of the isolates were mutant types following EUCAST ECOFFS.

#### MICs of CIP

Overall, the range of MICs of CIP of the investigated isolates was 0.047 - > 32 μg/ml. MIC 50 and 90% of all the tested isolates were 0.38 and > 32 μg/ml for CIP. More detailed information concerning MICs of CIP of the isolates can be found in Additional file 3.

Using Clinical and Laboratory Standards Institute (CLSI) MIC breakpoints, 126 isolates were susceptible to CIP (49.6%). G3 (FQ-treated piglets) showed the highest proportion of FQ-resistant isolates, with CIs not overlapping and indicating significance. Only seven FQ-resistant isolates were observed in G5, two isolates showed resistance against CIP (MIC = 3 and 4 μg/ml). One of the 254 isolates was determined as wild-type following EUCAST definition. Further information can be obtained from Table 4.

**Table 4:**
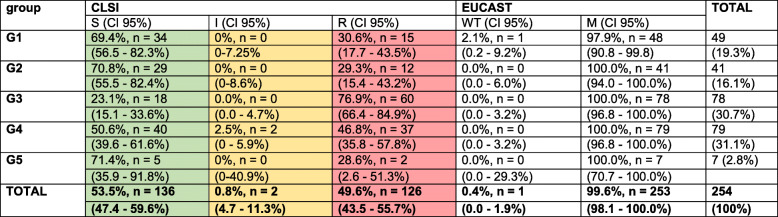
Faecal samples: Proportions of ciprofloxacin intermediate and resistant (CLSI) and wildtype and mutant (EUCAST) isolates, respectively

Ciprofloxacin MICs interpretation: Proportions (%) and the corresponding 95% confidence intervals in brackets (). n = number of strains. Light green, yellow and red areas indicate the susceptible, intermediate and resistant isolates according to the CLSI guidelines 2020 for human breakpoints. WT and M indicate the numbers of strains classified as wildtype or mutant strain according to the EUCAST guidelines 2021

#### NA and CIP susceptibility proportions (groups and age categories)

No significant differences could be observed in terms of the proportions of susceptibility of the investigated isolates among the study groups and within specific time points, except weaners from G2 and G3, where CIs of CIP MICs did not overlap (weaners G2: 100% (6/6) isolates susceptible, CI 66.9–100%; weaners G3: 05 (0/4) isolates susceptible, CI 0.0–44.5%).

## Discussion

### Proportions of detectable QNSE in faecal samples

The highest proportion of samples with detectable QNSE was found in treated pigs (G3). Further, following CLSI definitions it was the group with the significant highest number of FQ-resistant *E. coli* isolated. Our results support other studies, which showed that FQ resistance is positively associated with previous FQ treatment and can be reduced through restricted FQ use [29, 30].

Concerning QNSE in faeces of piglets pertaining to FQ-treated sows (G1), from piglets to weaners the proportion of samples with QNSE decreased significantly in this group. Similar results were observed in a French study investigating flumequine-treated sows and their progeny from various farrow-to-finish herds. However, in the respective study, from weaners to finishers the percentage of quinolone-resistant *E. coli* was still decreasing [31]. This contrasts with our study revealing a higher proportion of samples with QNSE in fattening pigs compared to weaners but with overlapping 95% CIs. To the author’s knowledge, this is the first report describing an increase of QNSE detected in fattening pigs.

In our study pigs, no additional FQ treatments were given except for two weaners which received a second FQ treatment. According to our enquiries, the other animals with which our study animals were grouped together in the fattening units had previously had received FQ treatment on the breeding farm. Thus, we assume that these pigs may have also spread QNSE, with the herd mixing resulting in the increase in QNSE positive study pigs. Interspecies transfer of NA-resistant *E. coli* in chickens and cattle has been published before [32, 33].

No significant differences among study groups could be observed in terms of the proportion of samples with QNSE at any specific sampling time (i.e. at the four different ages from farrowing to fattening unit). This was rather surprising to us as we assumed that different FQ backgrounds (treated vs. contact vs. control animals) would lead to different proportion of samples with detectable QNSE. The relatively low number of sampled pigs per group and age might have concealed this effect and could be rechecked by larger group designs.

All QNSE isolates of the control group originated from pigs from the same farm that had reported the last FQ treatment to be 3 months before sampling. In the other control farms, no QNSE were found and the last FQ treatment was reported between 30 and 34 months before sampling. This contrasts with a Swedish and English study finding quinolone-resistant bacteria isolated in swine without any prehistory of FQ use [34, 35]. Considering our study results and the long half-life of FQ, only a prolonged absence of fluoroquinolone treatment in pig farming likely leads to a reduced frequency of fluoroquinolone resistance (FQR) in the farm environment.

Although results must be compared with caution because of different material and methods performed, proportion of samples with QNSE were markedly higher in our study compared to Belloc et al. [31]. Neonatal antimicrobial treatment was reported to have a negative influence on microbial diversity. It decreases the abundance of protective commensal bacteria which promotes the colonisation of antimicrobial-resistant bacteria [36]. This might explain the high proportion of samples with QNSE in our study piglets and the significant reduction in weaners.

QNSE were detected in both contact groups from piglets to fattening pigs having indirect or direct contact to treated pigs. Furthermore, at any age, the proportions of samples with QNSE in contact animals were sometimes equal or larger compared to treated pigs. Despite the small number of study animals (*n* = 15) and use of a different indicator bacterial species (*Campylobacter*), comparable proportions of FQ-resistant *Campylobacter* in contact animals were discovered in a Japanese study [37]. This contrasts to a German experimental trial where four CIP-resistant *E. coli* isolates were detected in each treated and contact group but 47 CIP-resistant *E. coli* isolates in the control group. Possible factors explaining the low proportions of CIP-resistant *E. coli* isolates in treated and contact animals in the study include the experimental environment conditions (free of CIP-resistant *E. coli*), choice of study animals (single breeding unit, no previous antimicrobial use in sows and piglets), high hygiene and biosecurity standards during the study and small group sizes. Contact animals either become colonised by FQ-resistant bacteria by oral uptake via faeces and urine or antimicrobial residues in excretions which exert a selection for FQ-resistant bacteria [19]. Separation of diseased animals and proper hygiene levels are keys to promote healing and prevent infectious diseases from spreading in animal herds [38]. According to the above results, this measurement is also advisable to reduce the risk of transferring resistant bacteria or exposing animals and human beings to antimicrobial residues.

### Counts of QNSE per gram faeces

In our study, with increasing age of the pigs, we detected continuously decreasing mean and median counts of QNSE per gram faeces. Samples from 2 weeks old FQ-treated piglets or piglets of treated sows showed the highest counts of QNSE which met our expectations. Treated pigs were shown before to have higher counts per gram faeces dependent on dosing than placebo pigs [39]. QNSE - counts of piglets from treated sows were lower compared to those of treated piglets, leading us to the hypothesis that they received a lower dose of FQ via milk than treated piglets. Nonetheless, the number of QNSE detected in these samples is still considerable even with the treatment of piglets and sows being approximately ten to 14 days ago.

In a study measuring FQ-resistant *Campylobacter* in weaners (age: 18 days) during and after FQ administration, similar amounts (10^5^ to 10^7^ colony forming units per gram faeces) were found 5 days post-treatment [37]. In our study, counts in samples from 4 weeks old piglets were tenfold higher in piglets of treated sows and corresponding contact piglets compared to treated piglets and corresponding contact piglets. The quantity of FQ used are dosed according to the animal’s body weight. Consequently, in farms with FQ treatment in sows, larger quantities of FQ were used. This may lead to more residues in milk, faeces and the environment, which could explain these high counts in 4 weeks old piglets.

In a Swedish study, successful vertical transmission of quinolone-resistant *E. coli* was described in broiler production by introducing positive breeding birds [40]. In a recent study, piglets of sows where ampicillin or azithromycin resistance had been detected had a higher chance of being positive for these resistances [41]. This means that transmission of QNSE from positive sows to their progeny could explain the high proportions of samples with QNSE from piglets of treated sows and corresponding contact piglets in our study.

In the count part of the hurdle model, contact weaners (Ctat) had significantly higher counts compared to treated weaners. A similar outcome was observed in a study, where higher amounts of FQ-resistant *Campylobacter* were detected in untreated contact weaners compared to a FQ treated weaner housed in the same pen [37]. These results lead to the assumption that QNSE could be maintained and lingers better in the intestinal floras of contact weaners than in those of treated weaners. The intake of antimicrobial residues and therefore the exposure of the intestinal flora to low doses of antimicrobials could promote the development of resistant bacteria or exchange of antimicrobial resistance genes between bacteria in contact animals.

In agreement with other investigations, in both weaners and fattening pigs, the results of the hurdle model showed significantly lower counts in control animals compared to treated and contact animals in our study [37]. An explanation for this finding may be, as already mentioned, that our study was carried out under field conditions with lower hygienic standards compared to the other experimental studies. Furthermore, no significance was found in the zero part of the hurdle model among the groups (Trt, Ctat and Ctrl) and between weaners and fattening pigs. However, the count part of the hurdle model showed that there is a significant difference in counts of QNSE between weaners and fattening pigs and among treated, contact and control group. Differences in the zero part and the count part could be explained by a high proportion of samples where no QNSE could be found.

### MIC of QNSE isolated from faeces

Looking at the distribution of CIP-resistant *E.coli* strains, most of them were found in pigs which were part of treated pigs and the corresponding contact pigs.

Resistance proportions markedly differed between the group of directly treated pigs and the other study groups. This agrees with the results of Römer et al. (2017) comparing *E. coli* growth on enrofloxacin-supplemented agar plates between an experimental and control group although MIC values of the two groups did not differ significantly [18]. In the study of Römer et al. (2017), the experimental group was held in the same room as the control group which only tested positive for non-wildtype-*E. coli* (agar with 0.125 mg/L enrofloxacin) after the second treatment at day 28 and for enrofloxacin-resistant *E. coli* (agar with 4 mg/L enrofloxacin) at day 42 [18]. On the contrary in our study, we found QNSE- and FQ-resistant *E. coli* in both treated and contact piglets at 2 and 4 weeks of age. This emphasises that transmission may be faster under field conditions, e.g. by higher animal density, compared to laboratory standards.

CIP-resistant strains showing high MICs were detected in faeces of pigs of almost all groups and age categories of our trial but most of them were collected from 2 and 4 weeks old piglets. Similar peak times were observed by Delsol et al. (2004) and Belloc et al. (2005) [31, 42]. In the study of Burow et al. (2018) detection times were different. First CIP non-wildtype *E.coli* isolates were detected at day 56 (app. 7 weeks after treatment) in orally treated pigs and their contact pigs [19]. Surprisingly, control animals were tested positive much earlier from day one up to 42 days after treatment in that study.

Treated pigs having a higher risk of carrying CIP-resistant strains, agreed with our expectations after assessing the current literature [18, 39, 41, 43]. Further, we would have expected control pigs to have significantly different susceptibility proportions compared to treated piglets or their corresponding contact piglets. The low number of strains isolated in the different groups of weaners and fattening pigs may have made it impossible to observe such a difference. Other studies with more strains per group are needed to test differences in susceptibility proportions.

### Limitations of the study

Sampling was performed after a recently introduced law revision concerning more restrictive requirements for veterinary prescriptions of FQ on pig farms in Switzerland [12]. Therefore, only a small number of farrowing units were left that met our inclusion criteria which could have caused a selection bias. In previous studies investigating FQ resistance in pigs, animal husbandry and FQ treatment were managed by the study investigators, which is why we chose our study to be carried out under field conditions [18, 19, 44]. This included FQ treatment performed by the individual farmers who used different FQ products and different prescriptions (single versus multiple treatments) according to their own veterinarian. In terms of antimicrobial treatments, a distinction was only made between treatments of sows and piglets in the groups, but not the treatment frequency and thus the amount of fluoroquinolone administered in each case. In future studies, the influence of the quantity of fluoroquinolones administered on the quantitative and qualitative emergence of QNSE should also be investigated.

Treated and contact animals were either held in the same pen or room. Besides other farm-specific effects, these differences could have impacted our results, e.g. different treatment schemes could lead to variable proportions of QNSE in total *E. coli*, counts of quinolone non-susceptible *E. coli* and MICs of CIP.

Only the influence of FQ treatment on the occurrence of commensal QNSE in the different experimental groups was investigated in the present study. In future, it should be investigated whether the observations made in this study also apply to pathogenic bacteria, which could have an impact on treatment efficacy when using FQ in pigs.

Treatment of sows or piglets was mostly performed shortly after birth due to PPDS. Collecting necessary amounts of faeces from newly born piglets for the laboratory methods used is almost impossible. Due to these limitations control sampling of piglets before treatment was not performed. Taking rectal swabs would be an alternative for future projects [19]. Different methods for testing of G1 and G2 piglets (pooled samples) versus G3 and G4 (individual samples) could have influenced the identification of differences among these groups concerning quantitative detection and proportions of QNSE-positive samples at specific time points. For the investigation of the MICs of the selected isolates, we do not expect any influence of sample type.

The specific mode of operation in a sow-pool-system, where sows are transported between farms, may have contributed to the spread of resistant bacteria within some of the study herds.

Evidence of import and vertical transmission of quinolone resistant *E. coli* in hatcheries without antimicrobial selective pressure was recently published [40]. Enrofloxacin and CIP concentrations were measurable in blood serum samples of control weaners grouped with FQ orally and parenterally treated weaners [19]. Thus, it remains unclear if the source of QNSE in piglets without direct FQ treatment were the sows or the accompanying-treated piglets (transmitting QNSE via birth and excretions) or a selective pressure made by FQ residues (excreted via milk, urine and faeces) in their environment.

After freezing (− 80 °C, swabs stored in tryptic soy broth and glycerol) recovery rate of FQ-susceptible and resistant *E. coli* was reported to be good but with a significant reduction in number of *E. coli* at a storage temperature of - 20 °C (faecal slurries with phosphate-buffered saline and glycerol) [45, 46]. In our study faeces were stored in stool tubes at − 20 °C because of subsequent processing. According to these two reports, we expect that there was a quantitative reduction of *E. coli* in our study. The mildly selective medium used (Rapid-*E. coli* 2 agar plates supplemented with 8 mg/L NA) and picking one isolate of each sample might have distorted our results by over or underestimating the detection of QNSE.

## Conclusion

Quinolone-non-susceptible *E. coli* were shown to be widespread in the study farms. The occurrence of QNSE and FQ-resistant bacteria in the study pigs was associated with FQ use in farrowing units but was also observed in both contact and control animals. It is evident that through horizontal transfer there are no boundaries to QNSE and FQ-resistant bacteria when it comes to contact animals and the environment. Further, restricted or non-use of FQs is not the only measure required to minimise or eliminate QNSE in pig farming. Further research on the spread of QNSE and its promoting factors are necessary. Adapting a special management of antimicrobial-treated pigs in farms, restricted transport and purchase are also of concern.

## Supplementary Information

**Additional file 1. **Minimal inhibitory concentrations (MICs) of nalidixic acid and ciprofloxacin and counts of colony forming units of 254 randomly selected *Escherichia coli* isolates of faecal samples of pigs of G1-G5 at different ages

**Additional file 2. **Minimal inhibitory concentrations (MICs) and MIC 50 and 90% of nalidixic acid of 254 randomly selected *Escherichia coli* isolates of faecal samples of pigs of G1-G5 at different ages

**Additional file 3. **Minimal inhibitory concentrations (MICs), MIC 50% and MIC 90% of ciprofloxacin of 254 randomly selected *Escherichia coli* isolates of faecal samples of pigs of G1-G5 at different ages

## Data Availability

The datasets used and/or analysed during the current study are available from the corresponding author on reasonable request.
