# Supplementary material for: Occurrence of Escherichia coli non-susceptible to quinolones in faecal samples from fluoroquinolone-treated, contact and control pigs of different ages from 24 Swiss pig farms
Source: Porcine Health Manag. 2021 Apr 2;7:29. doi: 10.1186/s40813-021-00209-y (PMC8017651; doi:10.1186/s40813-021-00209-y)
Supplement: Supplementary file 1 — Additional file 1. Minimal inhibitory concentrations (MICs) of nalidixic acid and ciprofloxacin and counts of colony forming units of 254 randomly selected Escherichia coli isolates of faecal samples of pigs of G1-G5 at different ages [file 40813_2021_209_MOESM1_ESM.pdf]

Isolate	Unit	Group	QNSE CFU/g or ml	QNSE log CFU/g or ml	MIC_{NA}	MIC_{CIP}
1	Farrowing	1	32’000’000	7.5	256	0.125
2	Farrowing	1	42’000’000	7.6	256	0.125
3	Farrowing	1	1’330’000	6.1	96	0.094
4	Farrowing	1	970’000	6.0	>256	>32
5	Farrowing	1	10’480’000	7.0	>256	>32
6	Farrowing	1	14’880’000	7.2	256	0.125
7	Farrowing	1	4’180’000	6.6	256	0.125
8	Farrowing	1	4’420’000	6.6	256	0.19
9	Farrowing	1	680’000	5.8	128	0.125
10	Farrowing	1	3’160’000	6.5	256	0.19
11	Farrowing	1	28’800’000	7.5	96	0.125
12	Farrowing	1	6’130’000	6.8	192	0.125
13	Farrowing	1	16’160’000	7.2	128	0.125
14	Farrowing	1	180’000	5.3	>256	3
15	Farrowing	1	2’000’000	6.3	96	0.125
16	Farrowing	1	3’440’000	6.5	96	0.125
17	Farrowing	1	21’680’000	7.3	128	0.125
18	Farrowing	2	85’000	4.9	>256	6
19	Farrowing	2	1’500’000	6.2	256	0.19
20	Farrowing	2	70’000	4.8	256	0.125
21	Farrowing	2	1’000	3.0	>256	>32
22	Farrowing	2	47’000	4.7	128	0.125
23	Farrowing	2	1’000	3.0	256	0.125
24	Farrowing	2	3’940’000	6.6	96	0.094
25	Farrowing	2	450’000	5.7	>256	>32
26	Farrowing	2	23’280’000	7.4	>256	>32
27	Farrowing	2	260’000	5.4	192	0.125
28	Farrowing	2	220’000	5.3	>256	4
29	Farrowing	2	3’220’000	6.5	128	0.125
30	Farrowing	2	24’000’000	7.4	96	0.125
31	Farrowing	2	87’000	4.9	>256	4
32	Farrowing	2	4’880’000	6.7	64	0.094
33	Farrowing	2	720’000	5.9	192	0.19
34	Farrowing	2	1’380’000	6.1	192	0.125
35	Farrowing	2	35’000’000	7.5	>256	3
36	Farrowing	3	35’000	4.5	>256	8
37	Farrowing	3	900	3.0	24	0.125
38	Farrowing	3	43’200’000	7.6	>256	8
39	Farrowing	3	1’556’000	6.2	>256	>32

Isolate	Unit	Group	QNSE CFU/g or ml	QNSE log CFU/g or ml	MIC_{NA}	MIC_{CIP}
40	Farrowing	3	100’000	5.0	>256	8
41	Farrowing	3	72’800’000	7.9	>256	8
42	Farrowing	3	3’700’000	6.6	>256	>32
43	Farrowing	3	7’000	3.8	>256	>32
44	Farrowing	3	1’000’000	6.0	>256	8
45	Farrowing	3	700’000	5.8	>256	>32
46	Farrowing	3	1’000’000	6.0	>256	8
47	Farrowing	3	5’500’000	6.7	>256	>32
48	Farrowing	3	1’400’000	6.1	>256	>32
49	Farrowing	3	194’400’000	8.3	>256	>32
50	Farrowing	3	29’700’000	7.5	>256	8
51	Farrowing	3	1’600’000	6.2	>256	32
52	Farrowing	3	16’600’000	7.2	>256	>32
53	Farrowing	3	7’440’000	6.9	>256	24
54	Farrowing	3	12’300’000	7.1	>256	32
55	Farrowing	3	6’000	3.8	>256	32
56	Farrowing	3	4’000	3.6	>256	32
57	Farrowing	3	33’200’000	7.5	>256	24
58	Farrowing	3	34’400’000	7.5	>256	32
59	Farrowing	3	5’400’000	6.7	>256	32
60	Farrowing	3	62’400’000	7.8	>256	32
61	Farrowing	3	1’000’000	6.0	>256	8
62	Farrowing	3	3’500’000	6.5	>256	6
63	Farrowing	3	60’000	4.8	>256	8
64	Farrowing	3	100’000	5.0	>256	6
65	Farrowing	3	100’000	5.0	>256	4
66	Farrowing	3	200’000	5.3	256	0.125
67	Farrowing	3	140’000	5.1	>256	6
68	Farrowing	3	800’000	5.9	>256	6
69	Farrowing	3	100’000	5.0	>256	>32
70	Farrowing	3	40’000	4.6	>256	>32
71	Farrowing	3	50’000	4.7	>256	8
72	Farrowing	3	20’000	4.3	>256	>32
73	Farrowing	4	1’900’000	6.3	>256	8
74	Farrowing	4	1’870’000	6.3	256	0.19
75	Farrowing	4	30’000	4.5	>256	6
76	Farrowing	4	8’000	3.9	64	0.19
77	Farrowing	4	2’000	3.3	256	0.19
78	Farrowing	4	1’380’000	6.1	256	0.19

Isolate	Unit	Group	QNSE CFU/g or ml	QNSE log CFU/g or ml	MIC_{NA}	MIC_{CIP}
79	Farrowing	4	90’000	5.0	>256	>32
80	Farrowing	4	60’000	4.8	>256	0.38
81	Farrowing	4	3’000	3.5	>256	0.38
82	Farrowing	4	30’000	4.5	>256	>32
83	Farrowing	4	5’240’000	6.7	>256	>32
84	Farrowing	4	1’090’000	6.0	>256	>32
85	Farrowing	4	4’100’000	6.6	>256	>32
86	Farrowing	4	740’000	5.9	>256	8
87	Farrowing	4	60’000	4.8	>256	>32
88	Farrowing	4	40’000	4.6	256	0.094
89	Farrowing	4	6’080’000	6.8	256	0.19
90	Farrowing	4	1’440’000	6.2	256	0.19
91	Farrowing	4	110’000	5.0	256	0.125
92	Farrowing	4	20’000	4.3	256	0.094
93	Farrowing	4	100’000	5.0	256	0.094
94	Farrowing	4	800’000	5.9	256	0.094
95	Farrowing	4	30’000	4.5	>256	>32
96	Farrowing	4	20’000	4.3	>256	0.094
97	Farrowing	4	500’000	5.7	256	0.125
98	Farrowing	4	430’000	5.6	256	0.19
99	Farrowing	4	2’000	3.3	256	0.125
100	Farrowing	4	25’000	4.4	>256	12
101	Farrowing	4	330’000	5.5	256	0.125
102	Farrowing	4	1’000	3.0	256	0.125
103	Farrowing	4	40’000	4.6	>256	>32
104	Farrowing	4	25’000	4.4	>256	8
105	Farrowing	4	30’000	4.5	256	0.19
106	Farrowing	4	6’000	3.8	>256	6
107	Farrowing	4	7’000	3.8	>256	>32
108	Farrowing	4	440’000	5.6	>256	>32
109	Farrowing	4	40’000	4.6	>256	8
110	Farrowing	1	240’000	5.4	192	0.094
111	Farrowing	1	9’000	4.0	>256	0.125
112	Farrowing	1	220’000	5.3	>256	4
113	Farrowing	1	56’000	4.7	24	0.19
114	Farrowing	1	6’880’000	6.8	192	0.094
115	Farrowing	1	3’220’000	6.5	>256	32
116	Farrowing	1	1’480’000	6.2	192	8
117	Farrowing	1	61’000	4.8	>256	0.125

Isolate	Unit	Group	QNSE CFU/g or ml	QNSE log CFU/g or ml	MIC_{NA}	MIC_{CIP}
118	Farrowing	1	15’000’000	7.2	>256	>32
119	Farrowing	1	11’000	4.0	>256	3
120	Farrowing	1	3’440’000	6.5	96	0.094
121	Farrowing	1	119’000	5.3	256	0.125
122	Farrowing	1	89’000	4.9	>256	6
123	Farrowing	1	12’440’000	7.1	192	0.094
124	Farrowing	1	22’000	4.3	>256	3
125	Farrowing	1	60’000	4.8	>256	0.125
126	Farrowing	2	830’000	5.9	256	0.125
127	Farrowing	2	2’240’000	6.4	>256	0.094
128	Farrowing	2	3’840’000	6.6	96	0.094
129	Farrowing	2	500	2.7	>256	4
130	Farrowing	2	5’780’000	6.8	192	0.094
131	Farrowing	2	3’520’000	6.5	>256	32
132	Farrowing	2	4’000	3.6	128	0.125
133	Farrowing	2	4’240’000	6.6	128	0.094
134	Farrowing	2	5’120’000	6.7	>256	32
135	Farrowing	2	250’000	5.4	96	0.094
136	Farrowing	2	1’450’000	6.2	24	0.25
137	Farrowing	3	1’000’000	6.0	>256	0.19
138	Farrowing	3	2’976’000	6.5	>256	8
139	Farrowing	3	200	2.3	>256	>32
140	Farrowing	3	7’000	3.8	>256	6
141	Farrowing	3	224’000	5.4	48	0.19
142	Farrowing	3	74’000	4.9	>256	6
143	Farrowing	3	62’000	4.8	256	0.19
144	Farrowing	3	480’000	5.7	256	0.19
145	Farrowing	3	14’000	4.1	>256	9
146	Farrowing	3	59’000	4.8	>256	>32
147	Farrowing	3	1’000’000	6.0	256	0.19
148	Farrowing	3	1’000’000	6.0	>256	0.19
149	Farrowing	3	3’000	3.5	>256	>32
150	Farrowing	3	1’000’000	6.0	>256	6
151	Farrowing	3	300	2.5	256	0.125
152	Farrowing	3	200	2.3	>256	6
153	Farrowing	3	3’700	3.6	>256	6
154	Farrowing	3	69’000	4.8	>256	6
155	Farrowing	3	23’000	4.4	>256	6
156	Farrowing	3	30’000	4.5	>256	6

Isolate	Unit	Group	QNSE CFU/g or ml	QNSE log CFU/g or ml	MIC_{NA}	MIC_{CIP}
157	Farrowing	3	4’000	3.6	>256	6
158	Farrowing	3	11’000	4.0	>256	6
159	Farrowing	3	5’000	3.7	>256	6
160	Farrowing	3	784’000	5.9	>256	6
161	Farrowing	3	2’180’000	6.3	>256	6
162	Farrowing	3	33’000	4.5	>256	0.125
163	Farrowing	3	100	2.0	256	>32
164	Farrowing	3	800	2.9	>256	6
165	Farrowing	3	22’000	4.3	>256	6
166	Farrowing	4	269’000	5.4	>256	6
167	Farrowing	4	55’000	4.7	>256	6
168	Farrowing	4	1’000	3.0	>256	3
169	Farrowing	4	201’000	5.3	>256	6
170	Farrowing	4	21’000	4.3	>256	6
171	Farrowing	4	427’000	5.6	>256	6
172	Farrowing	4	2’000	3.3	>256	0.125
173	Farrowing	4	6’000	3.8	>256	6
174	Farrowing	4	5’000	3.7	>256	6
175	Farrowing	4	1’628’000	6.2	>256	>32
176	Farrowing	4	741’000	5.9	>256	>32
177	Farrowing	4	496’000	5.7	>256	0.25
178	Farrowing	4	57’000	4.8	>256	>32
179	Farrowing	4	19’000	4.3	256	0.19
180	Farrowing	4	50’000	4.7	256	0.125
181	Farrowing	4	4’000	3.6	256	0.125
182	Farrowing	4	18’000	4.3	256	0.19
183	Farrowing	4	1’000	3.0	256	0.125
184	Farrowing	4	400	2.6	256	0.19
185	Farrowing	4	1’000	3.0	256	0.125
186	Farrowing	4	400	2.6	256	0.19
187	Farrowing	4	200	2.3	256	0.125
188	Farrowing	4	14’000	4.1	256	0.19
189	Farrowing	4	31’000	4.5	>256	6
190	Farrowing	4	30’000	4.5	>256	6
191	Farrowing	4	2’000	3.3	>256	6
192	Farrowing	4	2’800	3.4	>256	6
193	Farrowing	4	6’000	3.8	>256	6
194	Farrowing	4	100	2.0	256	0.125
195	Farrowing	4	200	2.3	>256	6

Isolate	Unit	Group	QNSE CFU/g or ml	QNSE log CFU/g or ml	MIC_{NA}	MIC_{CIP}
196	Farrowing	4	148’000	5.2	>256	6
197	Farrowing	4	317’000	5.5	>256	6
198	Rearing	1	100	2.0	>256	0.19
199	Rearing	1	4’500	3.7	256	0.125
200	Rearing	1	300	2.5	24	0.047
201	Rearing	1	100	2.0	>256	6
202	Rearing	1	100	2.0	>256	4
203	Rearing	1	4’400	3.6	>256	0.125
204	Rearing	2	400	2.6	256	0.19
205	Rearing	2	400	2.6	256	0.19
206	Rearing	2	4’700	3.7	192	0.125
207	Rearing	2	100	2.0	256	0.125
208	Rearing	2	1’300	3.1	256	0.125
209	Rearing	2	39’600	4.6	192	0.125
210	Rearing	3	100	2.0	>256	>32
211	Rearing	3	100	2.0	>256	6
212	Rearing	3	100	2.0	>256	>32
213	Rearing	3	100	2.0	>256	32
214	Rearing	4	100’000	5.0	>256	6
215	Rearing	5	100	2.0	>256	3
216	Rearing	5	800	2.9	256	0.125
217	Rearing	5	100	2.0	>256	4
218	Rearing	5	200	2.3	>256	0.19
219	Rearing	5	1’100	3.0	>256	0.19
220	Fattening	1	3’700	3.6	>256	0.125
221	Fattening	1	100	2.0	256	0.19
222	Fattening	1	200	2.3	>256	8
223	Fattening	1	100	2.0	256	0.125
224	Fattening	1	900	3.0	>256	8
225	Fattening	1	15’000	4.2	256	0.125
226	Fattening	1	100	2.0	256	0.125
227	Fattening	1	100	2.0	>256	6
228	Fattening	1	400	2.6	192	0.19
229	Fattening	1	15’000	4.2	256	0.125
230	Fattening	2	100	2.0	>256	0.19
231	Fattening	2	500	2.7	256	0.19
232	Fattening	2	100	2.0	256	0.094
233	Fattening	2	7’100	3.9	>256	6
234	Fattening	2	100	2.0	>256	8

Isolate	Unit	Group	QNSE CFU/g or ml	QNSE log CFU/g or ml	MIC_{NA}	MIC_{CIP}
235	Fattening	2	100	2.0	256	0.125
236	Fattening	3	100	2.0	256	0.19
237	Fattening	3	200	2.3	256	0.19
238	Fattening	3	100	2.0	256	0.125
239	Fattening	3	100	2.0	256	0.19
240	Fattening	3	300	2.5	256	0.19
241	Fattening	3	200	2.3	256	0.19
242	Fattening	3	3’100	3.5	256	0.25
243	Fattening	3	200	2.3	256	0.125
244	Fattening	4	200	2.3	256	0.19
245	Fattening	4	500	2.7	256	0.25
246	Fattening	4	100	2.0	>256	0.19
247	Fattening	4	100	2.0	256	0.19
248	Fattening	4	600	2.8	256	0.19
249	Fattening	4	300	2.5	256	0.19
250	Fattening	4	100	2.0	256	0.125
251	Fattening	4	200	2.3	256	0.19
252	Fattening	4	2’500	3.4	256	0.19
253	Fattening	5	3’700	3.6	>256	0.125
254	Fattening	5	200	2.3	>256	0.19

QNSE CFU/g: quinolone non-susceptible *E. coli* counts in colony forming units per gram feces

QNSE log CFU/g: quinolone non-susceptible *E. coli* counts in log10 colony forming units per gram feces

MIC_{NA} : minimal inhibitory concentration of nalidixic acid in microgram per milliliter (µg/ml)

MIC_{CIP}: minimal inhibitory concentration of ciprofloxacin in microgram per milliliter (µg/ml)
