# Supplementary material for: Occurrence of Escherichia coli non-susceptible to quinolones in faecal samples from fluoroquinolone-treated, contact and control pigs of different ages from 24 Swiss pig farms
Source: Porcine Health Manag. 2021 Apr 2;7:29. doi: 10.1186/s40813-021-00209-y (PMC8017651; doi:10.1186/s40813-021-00209-y)
Supplement: Supplementary file 2 — Additional file 2. Minimal inhibitory concentrations (MICs) and MIC 50 and 90% of nalidixic acid of 254 randomly selected Escherichia coli isolates of faecal samples of pigs of G1-G5 at different ages [file 40813_2021_209_MOESM2_ESM.pdf]

group and age	concentrations of nalidixic acid in µg/ml								total	MIC 50%	MIC 90%
	24	48	64	96	128	192	256	>256			
**G1 all ages**	**2**	**0**	**0**	**5**	**3**	**6**	**13**	**20**	**49**	**256**	**>256**
G1 piglet2w	0	0	0	4	3	1	6	3	17	256	>256
G1 piglet4w	1	0	0	1	0	4	1	9	16	>256	>256
G1 weaners	1	0	0	0	0	0	1	4	6	>256	>256
G1 fattening pig	0	0	0	0	0	1	5	4	10	>256	>256
**G2 all ages**	**1**	**0**	**1**	**4**	**4**	**6**	**11**	**14**	**41**	**256**	**>256**
G2 piglet2w	0	0	1	2	2	3	3	7	18	256	>256
G2 piglet4w	1	0	0	2	2	1	1	4	11	192	>256
G2 weaners	0	0	0	0	0	2	4	0	6	256	256
G2 fattening pig	0	0	0	0	0	0	3	3	6	>256	>256
**G3 all ages**	**1**	**1**	**0**	**0**	**0**	**0**	**14**	**62**	**78**	**>256**	**>256**
G3 piglet2w	1	0	0	0	0	0	1	35	37	>256	>256
G3 piglet4w	0	1	0	0	0	0	5	23	29	>256	>256
G3 weaners	0	0	0	0	0	0	0	4	4	>256	>256
G3 fattening pig	0	0	0	0	0	0	8	0	8	256	256
**G4 all ages**	**0**	**0**	**1**	**0**	**0**	**0**	**35**	**43**	**79**	**>256**	**>256**
G4 piglet2w	0	0	1	0	0	0	16	20	37	>256	>256
G4 piglet4w	0	0	0	0	0	0	11	21	32	>256	>256
G4 weaners	0	0	0	0	0	0	0	1	1	>256	>256
G4 fattening pig	0	0	0	0	0	0	8	1	9	256	>256
**G5 all ages**	**0**	**0**	**0**	**0**	**0**	**0**	**1**	**6**	**7**	**>256**	**>256**
G5 weaners	0	0	0	0	0	0	1	4	5	>256	>256
G5 fattening pig	0	0	0	0	0	0	0	2	2	>256	>256
**TOTAL**	**4 (1.6%)**	**1 (0.4%)**	**2 (0.8%)**	**9 (3.5%)**	**7 (2.8%)**	**12 (4.7%)**	**74 (29.1%)**	**145 (57.1%)**	**254 (100%)**	**>256**	**>256**

Nalidixic acid MIC distribution by group and age: Numbers indicate the number of strains exhibiting the corresponding MIC value. MIC 50% and MIC 90% represent the concentration of nalidixic acid (µg/ml) inhibiting growth of 50% or 90% of strains, respectively. Piglet2w = two weeks old piglet, piglet4w = four weeks old piglet
