# Supplementary material for: Occurrence of Escherichia coli non-susceptible to quinolones in faecal samples from fluoroquinolone-treated, contact and control pigs of different ages from 24 Swiss pig farms
Source: Porcine Health Manag. 2021 Apr 2;7:29. doi: 10.1186/s40813-021-00209-y (PMC8017651; doi:10.1186/s40813-021-00209-y)
Supplement: Supplementary file 3 — Additional file 3. Minimal inhibitory concentrations (MICs), MIC 50% and MIC 90% of ciprofloxacin of 254 randomly selected Escherichia coli isolates of faecal samples of pigs of G1-G5 at different ages [file 40813_2021_209_MOESM3_ESM.pdf]

group and age	concentrations of ciprofloxacin in µg/ml														total	MIC 50%	MIC 90%
	S					I	R										
	0.047	0.094	0.125	0.19	0.25	0.38	3	4	6	8	12	24	32	>32			
**G1 all ages**	1	5	22	6	0	0	3	3	3	2	0	0	1	3	**49**	**0.125**	**8**
G1 piglet2w	0	1	11	2	0	0	1	0	0	0	0	0	0	2	17	0.125	>32
G1 piglet4w	0	4	4	1	0	0	2	2	1	0	0	0	1	1	16	0.19	>32
G1 weaners	1	0	2	1	0	0	0	1	1	0	0	0	0	0	6	0.19	6
G1 fattening pig	0	0	5	2	0	0	0	0	1	2	0	0	0	0	10	0.19	8
**G2 all ages**	0	8	14	6	1	0	1	3	2	1	0	0	2	3	**41**	**0.125**	**32**
G2 piglet2w	0	2	7	2	0	0	1	2	1	0	0	0	0	3	18	0.19	>32
G2 piglet4w	0	5	2	0	1	0	0	1	0	0	0	0	2	0	11	0.125	32
G2 weaners	0	0	4	2	0	0	0	0	0	0	0	0	0	0	6	0.125	0.19
G2 fattening pig	0	1	1	2	0	0	0	0	1	1	0	0	0	0	6	0.19	8
**G3 all ages**	0	0	6	11	1	0	0	1	19	12	0	2	9	17	**78**	**8**	**>32**
G3 piglet2w	0	0	2	0	0	0	0	1	4	10	0	2	7	11	37	24	>32
G3 piglet4w	0	0	2	6	0	0	0	0	14	2	0	0	1	4	29	6	>32
G3 weaners	0	0	0	0	0	0	0	0	1	0	0	0	1	2	4	>32	>32
G3 fattening pig	0	0	2	5	1	0	0	0	0	0	0	0	0	0	8	0.19	0.25
**G4 all ages**	0	5	13	20	2	2	1	0	18	4	1	0	0	13	**79**	**0.25**	**>32**
G4 piglet2w	0	5	5	8	0	2	0	0	2	4	1	0	0	10	37	0.38	>32
G4 piglet4w	0	0	7	5	1	0	1	0	15	0	0	0	0	3	32	6	32
G4 weaners	0	0	0	0	0	0	0	0	1	0	0	0	0	0	1	6	6
G4 fattening pig	0	0	1	7	1	0	0	0	0	0	0	0	0	0	9	0.19	0.25
**G5 all ages**	0	0	2	3	0	0	1	1	0	0	0	0	0	0	**7**	**0.19**	**4**
G5 weaners	0	0	1	2	0	0	1	1	0	0	0	0	0	0	5	0.19	4
G5 fattening pig	0	0	1	1	0	0	0	0	0	0	0	0	0	0	2	0.19	0.19
**TOTAL**	**1 (0.4%)**	**18 (7.1%)**	**57 (22.4%)**	**46 (18.1%)**	**4 (1.6%)**	**2 (0.8%)**	**6 (16.5%)**	**8 (3.1%)**	**42 (16.5%)**	**19 (7.5%)**	**1 (0.4%)**	**2 (0.8%)**	**12 (4.7%)**	**36 (14.2%)**	**254 (100%)**	**0.38**	**>32**

Ciprofloxacin MIC distribution by group and age: Numbers indicate the number of strains exhibiting the corresponding MIC value. Sensible (S), intermediate (I) and resistant (R) isolates, respectively. Breakpoints were obtained from the Clinical and Laboratory Standards Institute (CLSI) guidelines 2020 for human breakpoints. MIC 50% and MIC 90% represent the concentration of ciprofloxacin (µg/ml) inhibiting the growth of 50% or 90% of strains, respectively. Piglet2w = two weeks old piglet, piglet4w = four weeks old piglet
